# Patterns of mortality in public and private hospitals of Addis Ababa, Ethiopia

**DOI:** 10.1186/1471-2458-12-1007

**Published:** 2012-11-20

**Authors:** Awoke Misganaw, Damen Haile Mariam, Tekebash Araya, Kidane Ayele

**Affiliations:** 1 Addis Ababa Mortality Surveillance Program, College of Health Sciences, Addis Ababa University, Addis Ababa, Ethiopia; 2 College of Health Sciences, Addis Ababa University, Addis Ababa, Ethiopia

**Keywords:** Pattern, Mortality, Public Hospitals, Private Hospitals, Ethiopia

## Abstract

**Background:**

Ethiopia is encountering a growing burden of non-communicable diseases along with infectious diseases, perinatal and nutritional problems that have long been considered major problems of public health importance. This retrospective analysis was carried out to examine the mortality patterns from communicable diseases and non communicable diseases in public and private hospitals of Addis Ababa.

**Methods:**

Approximately 47,153 deaths were captured over eight years (2002–2010) in forty three public and private hospitals of Addis Ababa, Ethiopia. Data collectors (43 hospital clerks) and coordinators (3 nurses) had been extensively trained on how to review hospital death records. Information obtained included: dates of admission and death, age, sex, address, and principal cause of death. Only the diseases responsible for deaths are taken as the cause of death. Cause of death was coded using International Classification of Diseases (ICD-10) and data were double entered. Diseases were classified into: Group I (communicable diseases, maternal conditions and nutritional deficiencies); Group II (non-communicable causes); and Group III (injuries). Percentages, proportional mortality ratios, 95% confidence intervals (CI) and Adjusted odd ratios (OR) were calculated.

**Results:**

Overall, 59% of the deaths were attributed to Group I diseases, and 31% to Group II diseases and 12% to injuries. Nearly 56% of the males and 68% of the females deaths were due to five leading causes (conditions arising during perinatal period, HIV/AIDS, tuberculosis, cardiovascular diseases and respiratory infections). Significantly larger proportions of females died from Group I (67%) and Group II diseases (32%) compared with males (where the respective proportions were 52% and 30%). Significantly higher proportion of males (17%) than females (6%) were dying from Group III diseases. Deaths due to Group I diseases decreased while those due to Group II diseases increased with age. Overall Group I diseases and HIV/AIDS, tuberculosis and still birth mortality in particular have showed decreasing trend while Group II and III increasing over time. Double burden in mortality was highly observed in the age groups of 15–64 years. Those aged >45 years were dying more likely with non-communicable diseases compared with children. Children aged below 15 years were 16 times more likely to die from communicable, perinatal and nutritional conditions compared with elders. Mortality variation with age has been identified between public and private hospitals.

**Conclusions:**

The results of the present study shows that, in addition to the common Group I causes of death, emerging group II diseases are contributing to high proportions of mortality in the public and private hospitals of Addis Ababa, Ethiopia. Thus, priority should be given to the prevention and management of conditions arising during perinatal period such as low birth weight and still birth, HIV/AIDS; tuberculosis, respiratory infections, cardiovascular diseases, malignant neoplasm, chronic respiratory diseases and road traffic accident. The planning of health resources and activities should take into account the double burden in mortality due to Group I and Group II diseases. This calls for strengthening approaches towards the control and prevention of non-communicable diseases such as cardiovascular and malignant neoplasm.

## Background

In developing countries, there is a gradual decline of deaths from infectious diseases, while the proportion of deaths from non-communicable diseases is increasing [1,2]. Ethiopia is encountering a growing burden of non-communicable diseases (NCDs) along with those that have long been considered major problems of public health importance (infectious diseases, perinatal and nutritional problems) [3-5].

According to a recent estimate by the World Health Organization (WHO), NCDs are responsible for a third of the deaths in Ethiopia, while CVD, cancer, chronic respiratory diseases and diabetes mellitus contribute to a quarter [6]. The resulting double burden of diseases (the increase in NCDs, while pre-existing communicable diseases are still rampant), constrains the already meager health resources and hinders economic development [4,7,8]. The degree to which the current level of mortality might be reduced depends largely on the prevailing causes of death and the extent to which individual diseases may be prevented and controlled using different strategies [9].

Most of the mortality studies which attempted to describe the pattern of mortality in Ethiopia relied solely on hospital data. This was because of dearth of valid data on vital statistics as a result of inefficient system for national registration of deaths. Although hospital mortality may not be a true reflection of deaths from various causes in the general population, it may give insight into the burden of diseases in the community and may be valuable in evaluating health care delivery systems of the country. There have been reports of hospital mortality since the 1960s in Ethiopia [10]. However, the reports lack comprehensiveness, in particular in the past two and half decades when non-communicable diseases and the HIV pandemic might have significantly changed the disease pattern [10]. Therefore, this study is aimed at providing information on the current burden in causes of death in public and private hospitals over eight years in Addis Ababa, Ethiopia.

## Methods

### Study settings

This study was part of Addis Ababa Mortality Surveillance Program (AAMSP) the then project and in place since 2001 in Addis Ababa, Ethiopia. In Addis Ababa, totally there are 43 (21% of the total hospitals in the country) hospitals of which 12 are registered public and 31 are registered private hospitals. In addition, there are 41 health centers (24 governmental and 7 private) with 141 beds and 551 private clinics (109 special, 169 higher, 146 medium and 127 lower). Nearly 43% of the total medical doctors in the country are serving in these health facilities. The hospitals provide health care services not only for Addis Ababa residents but also serve as referral facilities for the nation [11,12]. According to the 2007 national census, the total population of Addis Ababa, the capital of Ethiopia was above 2.7 million, of which 47.6% were males and 52% were females. The total fertility rate of the city is below replacement level (1.5) and crude death rate is 9/1000. Infant mortality rate is 40/1000 [13] live births and maternal mortality rate is 0.001 [14].

### Study design

This retrospective study was carried out to examine the causes of all deaths in private and public hospitals in Addis Ababa over eight years period (2002–2010).

### Data collection procedures

There were 43 data collectors assigned within each hospital and three research coordinators. The data collectors and coordinators had prior relevant experience and extensive training on how to review hospital death records and registration books. To capture deaths in all hospitals, medical records and death registry books for the study period were reviewed. Information obtained includes: date of admission and date of death, name, age, sex, address, principal cause of death. Only the disease/s responsible for death is/are taken as the cause of death. Cause of death was coded according to the International Classification of Diseases, tenth revision (ICD-10) [15]. Approximately, 47,153 deaths were captured in public and private hospitals in Addis Ababa during the study period.

### Data management and analysis

Data were double entered to Access Microsoft Office spreadsheet and cleaned using STATA .do files. The 2006 Global Burden of Diseases classification was adapted to classify cause of deaths in our study. This classification categorized diseases into: Group I (communicable diseases, maternal conditions and nutritional deficiencies); Group II (non-communicable causes); and Group III (injuries) [16].

Percentages and proportional mortality ratios were calculated using STATA software. Binary logistic regression model was used to assess associations and significant differences, with adjusted odds ratio (OR) and 95% confidence intervals (CI).

### Ethical clearance

The program protocol was approved by Institutional Review Board (IRB) of the College of Health Sciences, Addis Ababa University, and the National Ethics Review of Committee of the Ethiopian Ministry of Science and Technology. Permission for the study had been also obtained from local authorities. In the office, individual information was accessible only to the research team and is kept confidential.

## Results

There were a total of 47,153 registered deaths; 41, 443 were public hospital and 5,710 were private hospital deaths, in the years 2002–2010 in Addis Ababa of which 57% (26,928) were those of males. The mean age of the deceased was 28 years (SD=23 years). Significantly larger proportions of females (67% and 32%) compared with males (52% and 30%) died from Group I and Group II diseases respectively. In contrast, significantly higher proportion of males (17%) than females (6%) died from Group III diseases (Table 1 &2).

**Table 1:** Cause of mortality distribution by gender in public and private hospitals, Addis Ababa, Ethiopia, 2002-2010

**Cause of Death**	**Number**	**Percent **^**a **^**(95% CI) **^**b**^		
		**Male**	**Female**	**Total**
**I**. **Group I**	**27**,**568**	**52** (**51**.**4** - **52**.**6**)	**67** (**66**.**4**-**67**.**6**)	**59** (**58**.**6**-**59**.**4**)
A. HIV/AIDS	5350	10 (9.6-10.4)	13 (12.5-13.5)	11 (10.7-11.3)
B. Tuberculosis	5,065	11	11	11 (10.7-11.3)
C. Respiratory Infections	3523	7	8	8 (7.8-8.2)
D. Diarrheal Disease	417	1	1	1
E. Meningitis	1139	2	2	2
F. Maternal Conditions	1299	0	6	3
G. Conditions arising during perinatal period	9337	17 (16.6-17.4)	24 (23.4-24.6)	20 (19.6-20.4)
- Low Birth Weight	2069	4	5	4
- Still Birth	7614	13 (12.6-13.4)	20 (19.4-20.6)	16 (15.7-16.3)
H. Nutritional Deficiency	876	2	2	2
**II**. **Group II**	**14**,**683**	**30** (**29**.**5**-**30**.**5**)	**32**(**31**.**4**-**32**.**6**)	**31** (**30**.**6**-**31**.**4**)
A. Malignant neoplasm	1,531	3 (2.8-3.2)	4 (3.7-4.3)	3 (2.8-3.2)
B. Diabetes Mellitus	801	2	2	2
C. Neuropsychiatric conditions	908	2	2	2
D. Cardiovascular diseases	5,375	11 (10.6-11.4)	12 (11.6-12.4)	11 (10.7-11.3)
Hypertensive heart disease	1047	2	2	2
Cerebrovascular disease	1223	3	3	3
Congestive Heart Failure	1267	2	3	3
Ischemic Heart Disease	1726	4	4	4
E. Respiratory diseases	3095	6 (5.7-6.3)	7 (6.6-7.4)	7 (6.6-7.4)
F. Digestive Diseases	2617	6	5	6
Chronic Liver Disease	987	2	2	2
G. Genitourinary Disease	1100	2	2	2
**III**. **Group III**	**5**,**776**	**17** (**16**.**6**-**17**.**4**)	**6** (**5**.**7**-**6**.**3**)	**12** (**11**.**7**-**12**.**3**)
A. Unintentional	2527	7	3	5
Road traffic accidents	1915	6 (5.7-6.3)	2(1.8-2.2)	4(3.8-4.2)
B. Intentional (Suicide…)	566	2	0	1
Total	47,153	100 (26,928)	100 (20,225)	100

* Totals may not add precisely.

^a^ Values above one rounded to nearest whole number and values less than one were not reported.

^b^ 95% confidence intervals have been provided for major categories and for the leading causes of death.

**Table 2:** Logistic regression for cause of deaths with age and sex in public and private hospitals in Addis Ababa, Ethiopia, 2002-2010

**Category**	**Number**	**Group I**	**Group II**	**Group III**
		**AOR (95% CI)**	**AOR (95% CI)**	**AOR (95% CI)**
**Sex**				
Male	26928	1	1	2.96 (2.76-3.18)
Female	20225	1.60(1.53-1.67)	1.24 (1.19-1.29)	1
**Age**				
0-14	15889	15.87 (14.55-17.31)	1	0.54 (0.48-0.62)
15-34	12742	2.89 (2.66-3.14)	2.05 (1.93-2.17)	2.57 (2.29-2.87)
35-44	6543	3.59 (3.28-3.93)	2.30 (2.15-2.46)	1.43 (1. 37–1.76)
45-54	4617	2.14 (1.94-2.35)	3.82 (3.55-4.11)	1.30 (1.13-1.51)
55-64	3435	1.28 (1.15-1.42)	6.25 (5.76-6.78)	1.13(0.97-1.33)
65+	3927	1	8.20 (7.57-8.87)	1

Abbreviations: AOR, adjusted odds ratio; CI, confidence Interval.

Adjusted for sex, age, years and type of institution (public, private).

Concerning specific diseases, higher proportions of females (24%) died from conditions arising during the perinatal period especially with still birth, 13% from HIV/AIDS, 12% from cardiovascular diseases and 7% from chronic respiratory diseases. Road traffic accidents accounted for higher proportions of male deaths (6%) compared with those of females (Table 1). Except maternal conditions, the other causes of deaths contributed nearly for equivalent proportions among males and females in private and public hospitals during the study period. Approximately 56% of the deaths among males and 68% of those among females were due to five diseases (i.e. conditions arising during perinatal period, HIV/AIDS, tuberculosis, cardiovascular diseases, respiratory infections).

**Figure 1:**
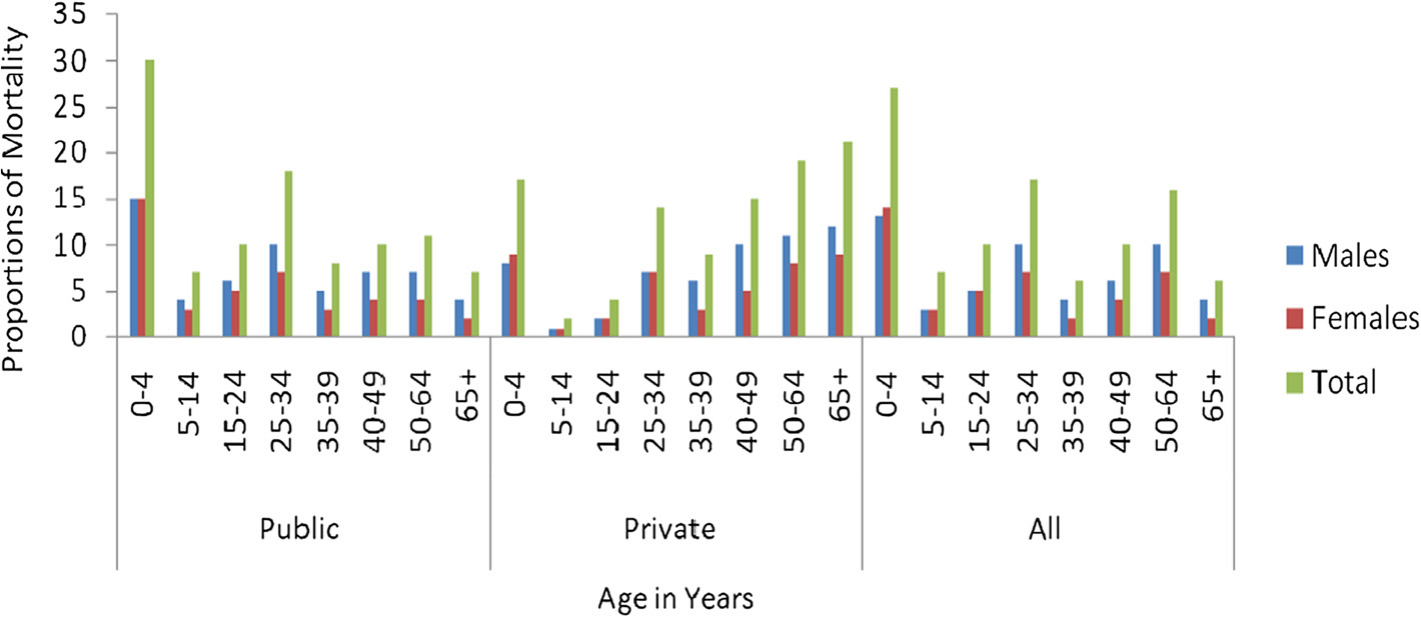
Mortality distribution by age and sex in public, private and all hospitals of Addis Ababa, Ethiopia, 2002–2010.

The distribution of total deaths by age and gender varies with hospital types. In public hospitals children aged 0–4 years and young adults aged 25–34 years accounted higher proportions, 30% and 18% of the total deaths respectively. In contrast, the higher proportions of deaths in private hospitals were elders aged 50–64 years and 65+ years accounted 19% and 21% respectively followed by children aged 0–4 years accounted for 17% (Figure 1). The distribution of the total deaths overall had three peaks: the first in childhood, the second in young adults and the third in old ages. Children aged 0–4 years accounted for 27% of the deaths and the proportion of males and females were equivalent. The second and third peaks were observed for young adults and adults aged 25–34 years and 50–64 years accounting for 17% and 16% of the total deaths respectively (Figure 1).

**Figure 2:**
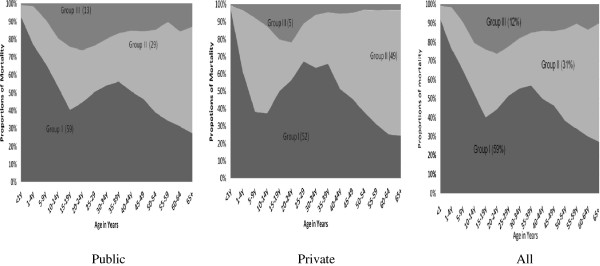
Causes of death by major categories and age in public, private and all hospitals in Addis Ababa, Ethiopia, 2002–2010.

Regarding causes of death, 59% of the total deaths in the private and public hospitals in Addis Ababa were attributed to Group I diseases, and 31% were to Group II diseases and 12% to injuries (Table 1 & Figure 2). For the age groups 0–14 years and 20–49 years, higher burden of Group I diseases were observed and for deaths aged above 40, higher burden of Group II diseases were observed. Higher deaths among young adults aged 15–40 years were observed due to injury (Figure 2). Deaths due to Group I diseases decreased significantly with age while those due to Group II increased. Double burden from Group I and Group II was highly observed in the age ranged 15–64 years (Figure 2 & Table 2). Higher proportions of deaths due to Group I, 13% of the total deaths, were observed in public hospitals than private hospitals of 5%; however higher proportions of Group II, 49% of the total deaths, were observed in private hospitals than public hospitals of 29% (Figure 2).

**Figure 3:**
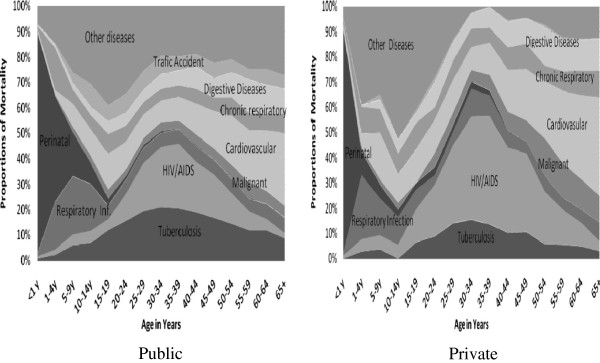
Proportions of deaths in public and private hospitals for the leading causes by age in Addis Ababa, Ethiopia, 2002–2010.

**Figure 4:**
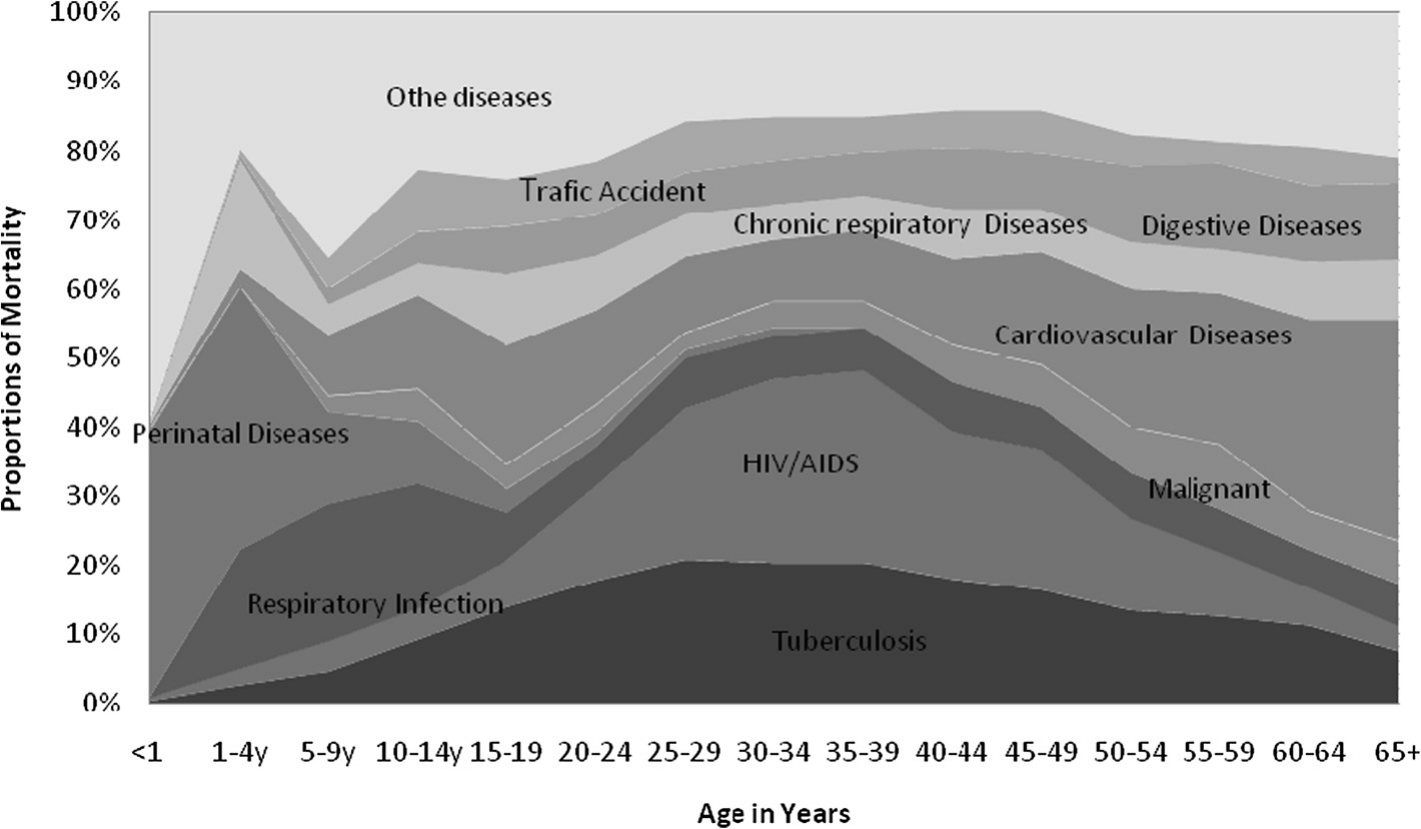
Proportions of deaths to all hospitals for the leading causes by age in Addis Ababa, Ethiopia, 2002–2010.

Comparing public and private hospitals on specific causes of death, higher proportions of the total deaths in private hospitals were from HIV/AIDS than public hospitals; however almost no traffic accident death observed in private hospitals (Figure 3). A limited number of diseases (diseases of perinatal conditions, HIV/AIDS, tuberculosis, CVD, respiratory infections, malignant neoplasm, chronic respiratory diseases, digestive diseases and road traffic accident) in the over all private and public hospitals of Addis Ababa accounted for more than 75% of the total deaths (Figure 4). For the age groups 0–14 years, the leading causes of deaths were mainly conditions arising during the perinatal period and respiratory infections. In the productive age groups 15–49 years, both HIV/AIDS and tuberculosis were the leading causes of death. Among adults with age 45 years and above, cardiovascular diseases and malignant neoplasm were among the leading causes of deaths (Figure 4). It had been observed that while age increased after 40 years, deaths due to HIV/AIDS and tuberculosis decreased but deaths due cardiovascular diseases increased.

Concerning the association of major category of diseases with gender and age among the different age groups, those aged 45–54, 55–64 and 65+ years were significantly more likely to die from non-communicable diseases with adjusted odd ratio 3.82, 6.25 and 8.20 respectively compared with children. In contrast, children aged 0–14 years were 16 times more likely to die from communicable, perinatal and nutritional conditions compared with those of aged 65 years and above (Table 2). On the other hand, people in the age group of 15–34 years were more likely to die from injury (AOR=2.57, CI, 2.29-2.87) (Table 2). In terms of gender, females were two times more likely to die from communicable diseases, perinatal, maternal and nutritional conditions and males were three times more likely to die from injury. Non-communicable diseases burden was observed to be slightly higher among females (Table 2).

**Figure 5:**
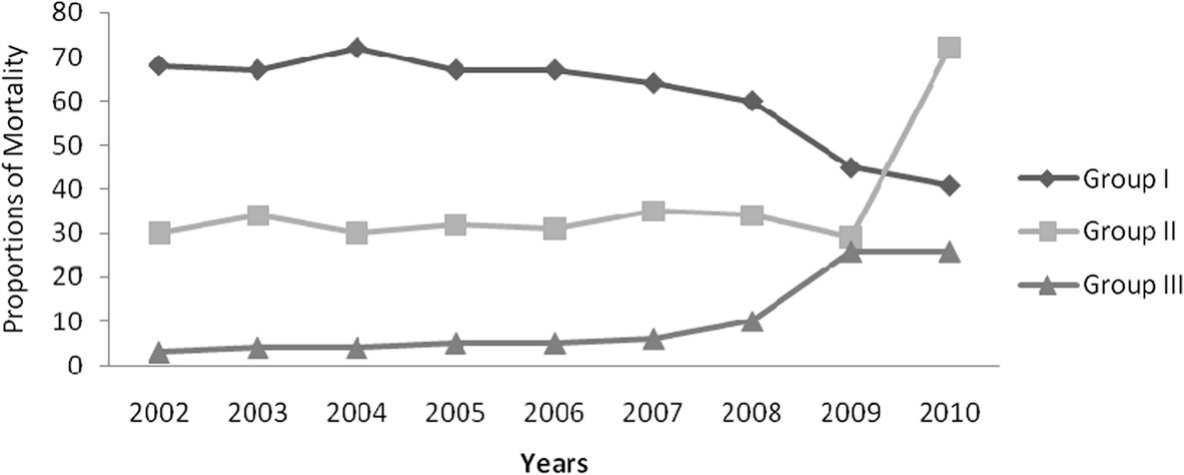
Trend of mortality from Group I, II and III diseases in all hospitals in Addis Ababa, Ethiopia, 2002–2010.

**Figure 6:**
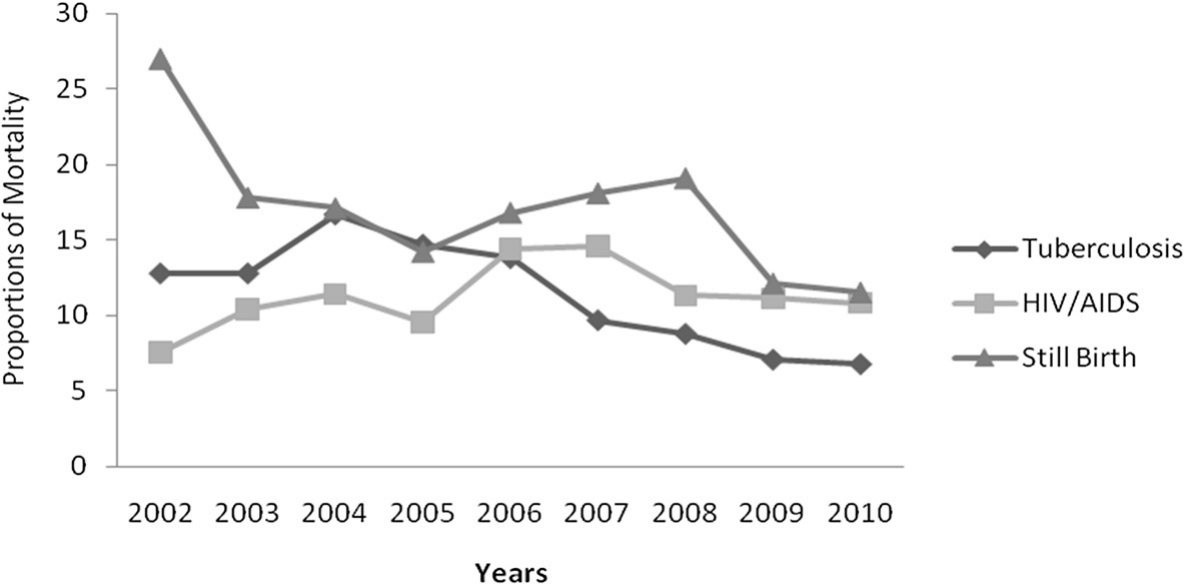
Trend of mortality from still birth, HIV/AIDS and Tuberculosis in all hospitals in Addis Ababa, Ethiopia, 2002–2010.

Group I disease caused mortality has decreased from 68% in 2002 to 41% in 2010. In contrast, Group III caused mortality has increased from 3% to 26% during the study period (Figure 5). As it can be seen from Figure 5, Group II caused mortality was almost the same until 2008 but increased from 30% to 71% afterwards. Concerning leading causes of deaths; HIV/AIDS caused mortality has increased from 9.5% in 2005 to 14.4% in 2006 then decreased to 10.8% in 2010. Similarly, tuberculosis caused mortality has decreased by half (Figure 6). Still birth has also decreased from 27% to 11.5% (Figure 6).

## Discussion

This study represent a very huge undertaken, and definitely provide very useful information both for internal use and for comparison with other countries in the region and beyond. We have collected mortality data for the period 2002–2010 in 43 public and private hospitals in Addis Ababa. We found that, of the over 47,000 deceased recorded, 59% infectious diseases, maternal and perinatal conditions, while 31% were due to non-communicable diseases and 12% to injuries. This findings support an underlying double burden of communicable and non-communicable diseases in the country [17].

In Ethiopia, health systems are straining under a double burden of persistently high communicable diseases coupled with non-communicable diseases [18,19]. The health systems were developed to provide care for infectious diseases and are, currently strived to attain Millennium Development Goals such as MDG 6. In line to this, decreasing trend of infectious diseases such as tuberculosis, HIV/AIDS in the study period may indicate the country is moving forward to MDG 6 [20]. However, the health systems were inadequately designed and resourced to care for people with chronic non-communicable diseases and injury related conditions where our analysis has showed the increasing trend on both groups.

In this context, hospital death records are an important source of data because they are readily available in the health facilities and allow mortality surveillance [17,21]. However, hospital based studies are prone to selection bias as patients admitted to the hospital may not be representative of all patients in the community and community wide inference should not be made from hospital data [17]. In addition, it was not possible to validate the data for accuracy or completeness of records.

Despite the limitations, results of this study can contribute to health policy in Ethiopia since the study covered many private and public hospitals in an urban setting where most health resources are concentrated [17]. Ethiopian health policy focus on maternal and child health, and communicable diseases such as tuberculosis and HIV/AIDS, these conditions are the leading causes of death in the country [22]. On the other hand, non-communicable diseases are also becoming the leading causes of death in the urban setting of Ethiopia and, therefore, require stronger policy attention [19]. Historically, infectious diseases have been the major causes of deaths in developing nations of the world [23][24] but, recently there are strong indications that deaths from infectious diseases are on the decline while non-communicable diseases are increasing [25].

This study has shown that more than a quarter of the deaths were children under the age of five years. This may reflect the venerability of this age group. In addition, conditions arising during perinatal period and respiratory infections were the major causes of death overall, which caused more than half of the mortality in this age group.

One of the leading causes of death, still birth has showed a reduction over eight years period in this analysis which may indicate Ethiopia is pushing towards achieving MDG 2 [13]. Infant and child mortality however remains disturbingly high in Ethiopia, despite the significant decline in most parts of the developing world [9]. This study has also showed higher proportions of under-5 mortality among public hospital deaths which may require further study and policy attention. Based on these findings, there is a need to further search for the possible reasons for high childhood mortality including high number female still births.

The majorities (50%) of the deaths were among young adults and adults aged 25–64 years which is the most economically productive segment of the society. This premature mortality from the double burden of communicable and non-communicable diseases has negative implication for the development of the nation.

According to the present findings, HIV/AIDS still remained the major cause of mortality followed by tuberculosis, cardiovascular diseases, respiratory infections and others. Since 2006, HIV/AIDS mortality has little decrement in public and private hospitals. This could be explained by the free antiretroviral therapy (ART) initiated in public and private hospitals of Addis Ababa in 2005 [26]. However, late diagnosis of the infection, poor drug adherence, possible drug resistance and societal attitude to the disease could be possible factors for the only little changes observed with HIV/AIDS mortality [27]. This and other reasons need to be investigated and addressed with public and private hospitals. In addition, this study has also showed higher proportions of HIV/AIDS mortality among private hospital deaths which may require further study and policy attention.

Non-communicable diseases like cardiovascular diseases and malignant neoplasm were identified among the leading causes of death in this study. In contrast to tuberculosis and HIV/AIDS, mortality from cardiovascular diseases and malignant neoplasm increased when age increased. This finding supports results of community based studies from Addis Ababa which reported cardiovascular diseases as being responsible for 24% [19] of the deaths while hypertensive conditions accounting for 30% of morbidity in another report [4]. This increase in non-communicable diseases is expected for the future especially in relation to "westernization" of our diet and life style changes in the urban setting of Ethiopia. This study has also showed higher proportions of non communicable disease mortality among private hospital deaths than public hospitals that may require further study and policy attention.

Most public health programs and local and international funds are vertical to address common diseases such as HIV/AIDS, TB, Malaria, Child and Maternal diseases [17]. It may require either integrating relevant programs and mobilize resource to highly prevalent diseases such as cardiovascular diseases, malignant neoplasm and injuries making resource equity. Monitoring the double burden in mortality is essential and the cost of obtaining health information has to be weighed against its usefulness. Using inputs based on readily available data may ensure sustainability of the information system and support evidence based health practice.

## Conclusions and recommendations

The results of the present study suggest that to reduce mortality in the urban setting of Ethiopia; priority should be given to the prevention and management of conditions arising during perinatal period such as low birth weight and still birth, HIV/AIDS, tuberculosis, respiratory infections, cardiovascular diseases, malignant neoplasm, chronic respiratory diseases and road traffic accident. The planning of health resources and activities should take in to account the double burden in mortality due to communicable diseases, perinatal conditions and non-communicable diseases. Approaches towards the control and prevention of non communicable disease conditions like cardiovascular and malignant neoplasm also deserve to be strengthened.

## Competing interests

The authors declare that they have no competing interests.

## Authors’ contributions

AM: he has made substantial contributions to conception and design, acquisition of data, analysis and interpretation of data, draft the manuscript and revising it critically for important intellectual content. DHM: he has made substantial contributions to conception and design, drafting the manuscript or revising it critically for important intellectual content and given final approval of the version to be published. TA: she has made substantial contributions to conception and design and involved in drafting the manuscript. KA: he has made substantial contributions to conception and design, drafting the manuscript or revising it critically for important intellectual content. All authors read and approved the final manuscript.

## Authors’ information

AM is a public health researcher in Addis Ababa Mortality Surveillance Program, College of health sciences, Addis Ababa University. He is a PhD candidate in public health, in the School of Public Health, Addis Ababa University. He has earned his Masters Degree in Public Health, School of Public Health, Addis Ababa University, Ethiopia. DHM is a public health professor in Addis Ababa University. Currently, he is senior researcher and Post Graduate and Research Associate Dean for College of Health Sciences, Addis Ababa University, Ethiopia. TA is Addis Ababa Mortality Surveillance Program Manager, College of Health Sciences, Addis Ababa University. She has earned PhD Degree in Public Health, from School of Public Health, Addis Ababa University, Ethiopia. KA is Addis Ababa Mortality Surveillance Assistant Researcher, College of Health Sciences, Addis Ababa University. He is a student in Masters Degree of social works, School of Social Works, Addis Ababa University. He has earned two Bachelor Degrees in Nursing and Law, Addis Ababa University, Ethiopia.

## Pre-publication history

The pre-publication history for this paper can be accessed here:

http://www.biomedcentral.com/1471-2458/12/1007/prepub
